# CNN-integrated ratiometric fluorescent tag for rapid monitoring of seafood freshness at refrigerated temperatures

**DOI:** 10.1016/j.crfs.2026.101560

**Published:** 2026-09-08

**Authors:** Wenyang Zhang, You Tian, Yanwu Chen, Min Wei

**Affiliations:** a College of Light Chemical Industry and Materials Engineering, Shunde Polytechnic University, Foshan, 528333, China; b School of Food Science and Engineering, South China University of Technology, Guangzhou, 510641, China; c Academy of Contemporary Food Engineering, South China University of Technology, Guangzhou Higher Education Mega Centre, Guangzhou, 510006, China; d Engineering and Technological Research Centre of Guangdong Province on Intelligent Sensing and Process Control of Cold Chain Foods, & Guangdong Province Engineering Laboratory for Intelligent Cold Chain Logistics Equipment for Agricultural Products, Guangzhou Higher Education Mega Centre, Guangzhou, 510006, China; e College of Food Science and Technology, Henan Key Laboratory of Cereal and Oil Food Safety Inspection and Control, Henan University of Technology, Zhengzhou, 450001, China

**Keywords:** Ratiometric fluorescent tag, Convolutional neural networks, Seafood freshness, Aggregation-induced emission, DPA-AgCuNCs

## Abstract

Given the perishable nature of seafood, accurate and rapid freshness monitoring is essential for ensuring food safety and public health. In this study, a ratiometric fluorescent tag was proposed for detecting ammonia to assess seafood freshness, using D-penicillamine-capped silver/copper nanoclusters (DPA-AgCuNCs) and fluorescein isothiocyanate (FITC) as dual-emission probes. The synthesized DPA-AgCuNCs with aggregation-induced emission (AIE) properties exhibited high sensitivity toward ammonia, with a limit of detection of 2.95 ppm (3σ/s). By integrating orange-emitting DPA-AgCuNCs with green-emissive FITC, the fabricated ratiometric fluorescent tag exhibited a distinct orange-to-green fluorescence transition with increasing ammonia concentration, enabling direct visual assessment of freshness. A fluorescence colour card was further established for rapid preliminary differentiation among fresh, less fresh, and spoiled samples. Convolutional neural network (CNN)-assisted image analysis was introduced as a complementary approach to improve the objectivity and consistency of freshness classification, achieving precision values of 95.24%, 100%, and 100% for fresh, less fresh, and spoiled shrimp, respectively. For on-site monitoring, the CNN was integrated into a smartphone application (FreshSense), constructing a simple platform for rapid monitoring of seafood freshness. This proof-of-concept system is rapid, on-site, and non-destructive, demonstrating its potential for practical screening in food quality and safety.

## Highlights

• DPA-AgCuNCs with AIE properties enable sensitive NH_3_ detection.
• A novel ratiometric fluorescent tag was developed based on DPA-AgCuNCs and FITC.
• ResNet-34 model achieved 98.36% accurate rapid seafood freshness prediction.
• CNN-integrated sensing enhances intelligent seafood quality evaluation.

## Graphical abstract

**Figure:**
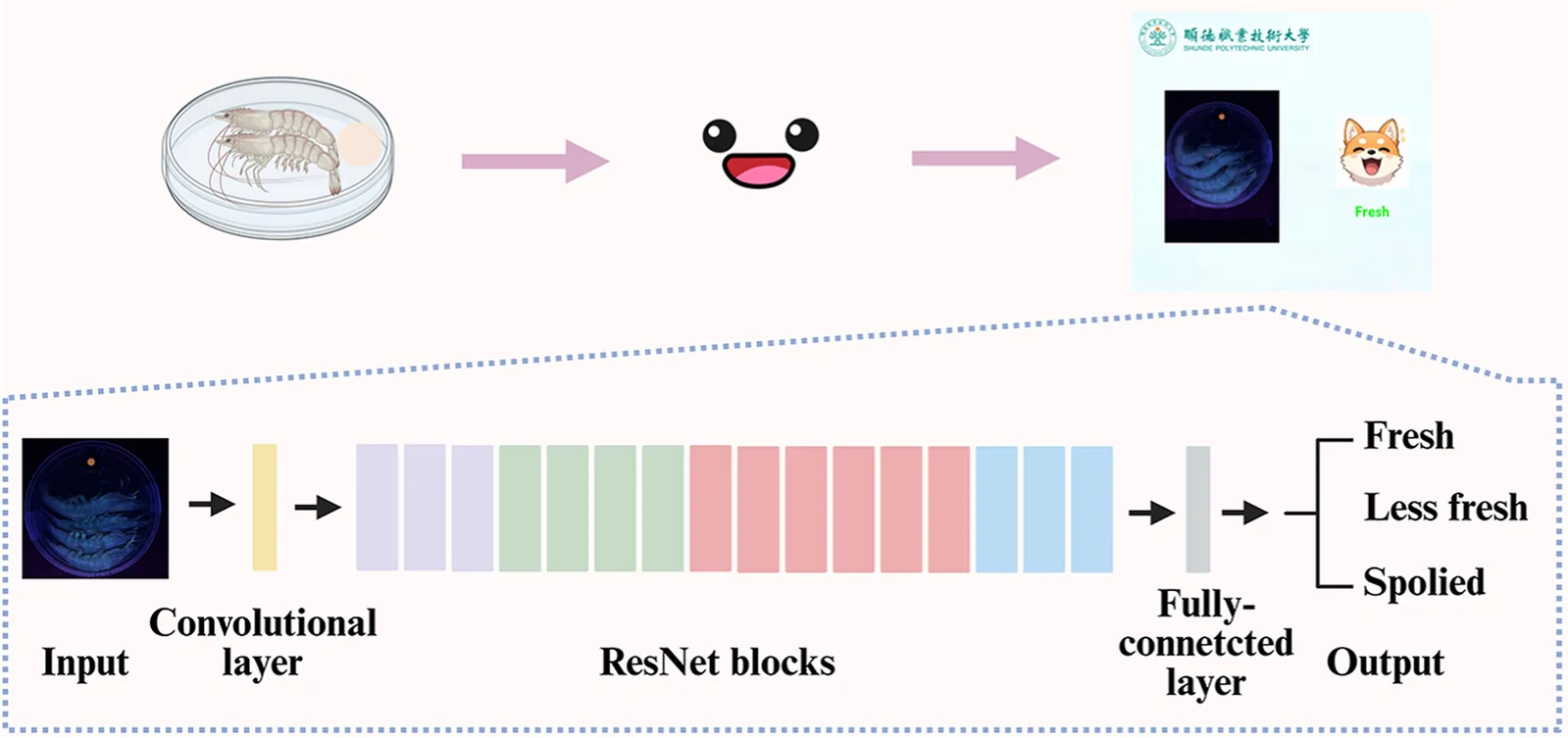

## 1 Introduction

Seafood is a vital source of nutrition in the human diet, and its freshness is crucial for both public health and consumer satisfaction (Dong et al., 2025; Hou et al., 2025). Low-temperature storage, including chilling and freezing, serves as the primary method for preserving fresh seafood, which can effectively inhibit bacterial growth and metabolic activity (Suárez-Medina et al., 2024). Nevertheless, even under refrigeration, the abundant nutrients in seafood render it highly susceptible to spoilage induced by endogenous enzymes and microorganisms, resulting in the release of volatile amines (VAs) (Jin et al., 2025). VAs, including ammonia (NH_3_) and biogenic amines (BAs), are key components of total volatile basic nitrogen (TVB-N) and serve as important indicators for monitoring of seafood freshness (Ouyang et al., 2026; Zhao et al., 2025). Although traditional analytical techniques, such as chemical-microbiological measurements (Song et al., 2024), chromatography (Yu et al., 2020), mass spectrometry (Muz et al., 2017), and spectroscopy (Yang et al., 2025), can accurately measure VAs, their dependence on time-consuming sample preparation and sophisticated instrumentation limits their application in the non-destructive, dynamic monitoring of seafood freshness (Ouyang et al., 2026).

Recent advances in non-destructive food quality assessment have highlighted the potential of portable fluorescence-based systems for real-time freshness monitoring (Gu et al., 2025; Wang et al., 2021). However, early fluorescence sensors based on single-signal response (off/on model) are susceptible to environmental interference and often lack sufficient visual contrast for reliable naked-eye detection (Miao et al., 2025; Zhang et al., 2022b). To overcome these limitations, ratiometric fluorescent probes, featuring two well-resolved emission bands, have been designed to provide intrinsic self-calibration and improve visual discrimination without external instrumentation (Guo et al., 2025). For instance, a carbon quantum dot (CQD)-based dual-emission probe was fabricated (Zhang et al., 2025), while a dual-emissive ratiometric fluorescent metal-organic framework (MOF) probe was also designed by covalently grafting fluorescein isothiocyanate (FITC) onto a lanthanide MOF (Wang et al., 2021). Although promising, many of these probes suffer from aggregation-caused quenching (ACQ) in aggregated form (Li et al., 2026). Fortunately, the emergence of aggregation-induced emission (AIE) provided a new strategy for designing ratiometric fluorescent probes (Cai and Liu, 2020). To date, only a few AIE-based ratiometric probes for VAs detection have been described (Zhang et al., 2022a), underscoring an urgent need to develop VAs-sensitive AIE probes.

As an emerging probe material, metal nanoclusters (MNCs) with high photostability, low biotoxicity, and tunable metal/ligand compositions have attracted significant attention in AIE research (Chang et al., 2024; Cheng et al., 2024). Over the past decades, extensive research has focused on fluorescent gold, silver, and copper monometallic nanoclusters (NCs), which have demonstrated considerable promise for rapid detection (Rival et al., 2021; Shi et al., 2021; Yue et al., 2022; Zhang et al., 2021). More recently, bimetallic NCs, defined as NCs incorporating two distinct metal elements within a single, well-defined structure, have garnered increasing attention due to their unique optical properties (Indongo et al., 2025). For instance, D-penicillamine (DPA) capped gold-copper NCs (Au/CuNCs) showed excellent sensitivity and selectivity toward NH_3_, making them an ideal AIE probe for monitoring of seafood freshness (Zhang et al., 2023). This was attributed to the synergistic effects generated by the bimetallic composition, which enhanced their stability and sensitivity during the detection of NH_3_. However, despite existing studies on various bimetallic NCs, such as AuCuNCs (Hu et al., 2025), AuAgNCs (Zhai et al., 2025), AgCuNCs (Indongo et al., 2025), and CuMnNCs (Tripathi et al., 2025), reports on those exhibiting AIE properties remain limited.

A defining feature of ratiometric fluorescent sensors lies in their ability to produce distinct colour transitions, enabling rapid and straightforward visual detection of targets. However, visual analysis is inherently subjective, owing to its reliance on the acuity of the operator. Individual differences in colour sensitivity may affect inconsistent interpretations (Hu and Yan, 2023). Therefore, utilizing automated colour-sensing technologies is crucial for overcoming limitations of human vision and ensuring the accuracy of monitoring. Encouragingly, artificial intelligence (AI), particularly convolutional neural networks (CNNs), has effectively resolved this dilemma. By extracting high-level features from complex multimodal data, AI provides a robust framework for objective signal interpretation and high-precision analysis (Ghorbanizamani, 2025; Liu et al., 2026b). AI-assisted sensors are no longer limited to simple detection functions; instead, they have comprehensive analysis capabilities, including spoilage classification and prediction. By enabling real-time, data-driven decision-making, these sensors effectively enhanced food safety and quality assurance standards (Liu et al., 2026a).

**Scheme 1:**
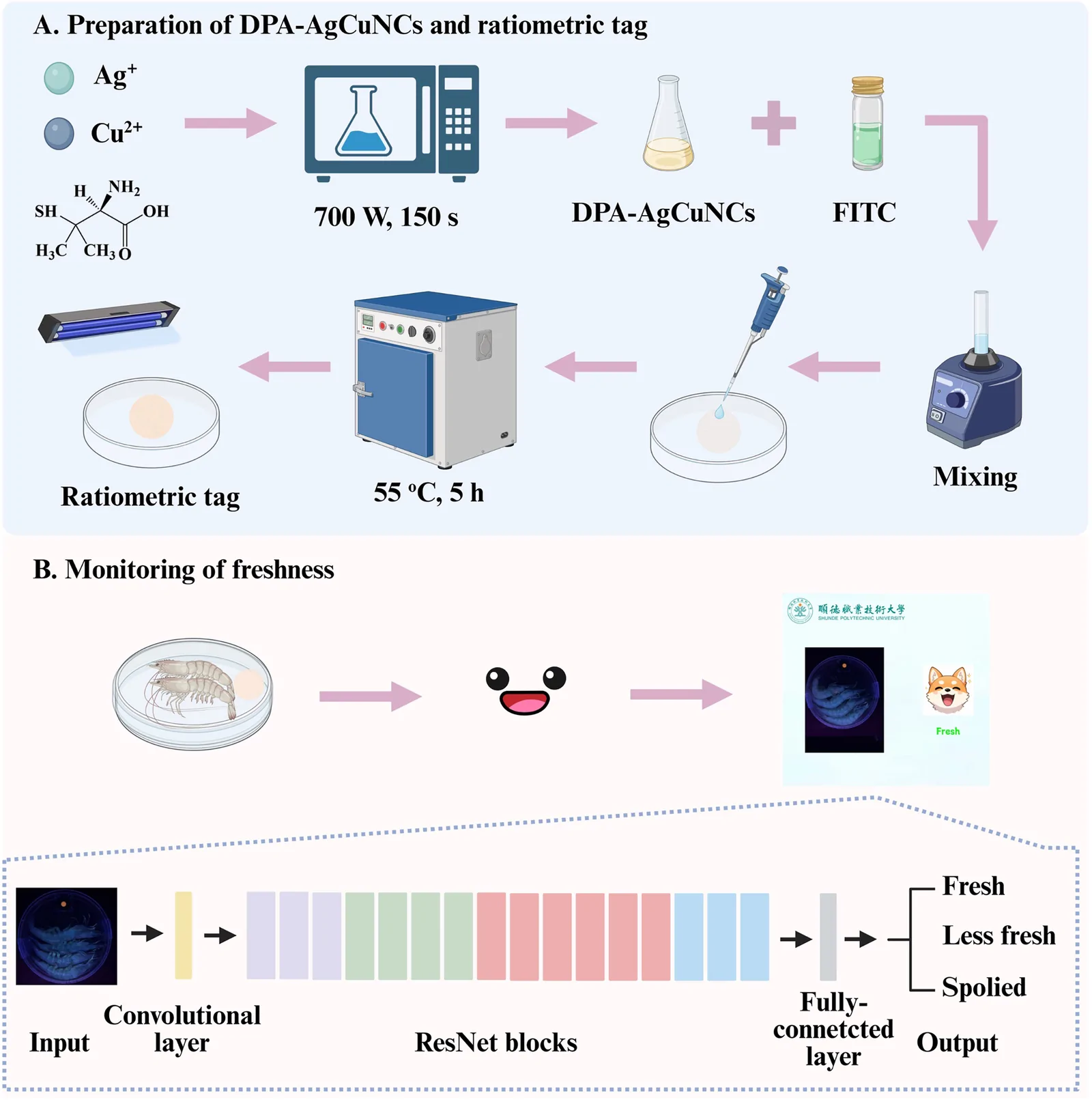
Schematic diagram of the proposed sensing platform: (A) preparation of DPA-AgCuNCs and ratiometric fluorescent tag; (B) a convolutional neural network (CNN)-assisted working principle for shrimp freshness identification.

In this study, a novel ratiometric fluorescent tag was developed by integrating D-penicillamine-capped silver/copper nanoclusters (DPA-AgCuNCs) and FITC as dual-emissive probes for rapid monitoring of VAs, particularly NH_3_, enabling effective seafood freshness evaluation (Scheme 1A). The sensing mechanism involved NH_3_-induced static-dynamic quenching of DPA-AgCuNCs and simultaneous pH-triggered fluorescence enhancement of FITC through the lactone-ring opening. This complementary response generated a distinct orange-to-green fluorescence transition, enabling direct visual assessment of freshness using a fluorescence colour card. To further improve the objectivity and consistency of freshness classification, CNN-assisted image analysis was introduced as a complementary approach, achieving an overall accuracy of 98.36% (Scheme 1B). The combination of visual ratiometric sensing and automated image analysis provides a flexible strategy for rapid seafood for rapid seafood freshness assessment and supports the further development of intelligent sensing platform.

## 2 Materials and methods

### 2.1 Materials, reagents, and apparatus

Fluorescein isothiocyanate (FITC), D-penicillamine (DPA), copper nitrate trihydrate [Cu(NO_3_)_2_·3H_2_O], sodium sulfide (Na_2_S), ammonium hydroxide solution (NH_3_·H_2_O, 25 wt%), methanol (MeOH), ethyl acetate (EtOAc), N, N-Dimethylformamide (DMF), dimethylamine (DMA), trimethylamine (TMA) putrescine (Put), dimethyl sulfoxide (DMSO), acetic acid (HAc), and formaldehyde solution (HCHO) were purchased from Aladdin Biochemical (Shanghai, China). Silver nitrate (AgNO_3_) was obtained from Sinopharm Chemical Reagent Co., Ltd. (Shanghai, China). All reagents were analytical grade and used without further purification. Fresh sand shrimp (*Metapenaeus ensis*) were bought from AEON supermarket in Foshan, China. Aqueous solutions were prepared using Milli-Q water (18.2 MΩ cm, Millipore, MA, USA).

Fluorescence spectra were recorded on a Thermo Scientific Lumina spectrophotometer (Thermo Fisher Scientific, Germany). Fluorescence lifetime spectra were performed on an Edinburgh FLS1000 spectrophotometer (Edinburgh Instruments Ltd., UK). Fluorescent images were captured under 365 nm UV light using an iPhone 16 Pro (Apple Inc., USA). Ultraviolet-visible (UV-vis) absorption spectra were obtained using a Thermo Scientific Evolution 220 UV-vis spectrophotometer (Thermo Fisher Scientific, Germany). The morphology, size, and elemental mapping of DPA-AgCuNCs were characterized by transmission electron microscopy (TEM) on a Talos F200X instrument (Thermo Fisher Scientific, Germany). The surface morphology of the loaded DPA-AgCuNCs/FITC ratiometric fluorescent tag was examined using Hitachi SU8010 scanning electron microscopy (SEM, Hitachi, Ltd., Japan). Fourier transform infrared (FTIR) spectra were collected using a Thermo Fisher Scientific Nicolet iN10 FTIR microscope (Thermo Fisher Scientific, Germany). X-ray photoelectron spectroscopy (XPS) analysis of DPA-AgCuNCs was conducted on a Thermo Scientific K-Alpha + spectrometer (Thermo Fisher Scientific, Germany). The zeta potential of DPA-AgCuNCs, with or without NH_3_, was measured using a Zetasizer from Nano ZS (Malvern Instruments Ltd., UK).

### 2.2 Synthesis of DPA-AgCuNCs

The synthesis of DPA-AgCuNCs was adopted from previous reported methods for DPA-AgNCs with appropriate modification (Jia et al., 2014). Typically, a solution of DPA (1.615 mmol) in 38 mL of ultrapure water was placed inside the 50 mL flask. Then, 2.4 mL of AgNO_3_ (0.1 M) and 0.6 mL of Cu(NO_3_)_2_ (0.1 M) were added to the above solution, stirring vigorously for 2 min. The mixture was heated in a microwave reactor system (Xiangedu Technology Co., Ltd., China) at 90°C and 700 W for 150 s. The resulting DPA-AgCuNCs were collected by centrifugation (7000 rpm, 10 min), washed twice with EtOH, and vacuum-dried at 70°C.

### 2.3 Fabrication of loaded DPA-AgCuNCs/FITC ratiometric fluorescent tag

The schematic illustration of the preparation of the loaded DPA-AgCuNCs/FITC ratiometric fluorescent tag (hereafter, the ratiometric tag) is shown in Scheme 1A. Briefly, 0.5 mL of FITC (100 μg/mL) and 4 mL of DPA-AgCuNCs (12 mM, calculated on an Ag-atom basis) were sequentially added into a 10 mL centrifuge tube. The mixture was homogenized by gentle vortexing for 5 min to ensure uniform dispersion. Then, 100 μL of the resulting ratiometric probe was drop-casted onto a 10-mm-diameter circular cellulose fiber paper. After drying in an oven at 50°C for 5 h, the ratiometric tag was obtained and stored at 4°C for further use. For comparative control experiments, the loaded FITC tag and the loaded DPA-AgCuNCs tag were fabricated following the same protocol, with ultrapure water substituted for DPA-AgCuNCs (4 mL) and FITC (0.5 mL), respectively.

### 2.4 Detection of ammonia gas

The NH_3_ detection experiment was performed in a sealed 2 L test chamber maintained at 25°C. The ratiometric tag was placed at the center of the test chamber. A precise volume of analyte was injected into the test chamber to establish a specific vapour concentration. The fluorescence colour changes of the ratiometric tag were acquired using a smartphone camera in a dark box under 365 nm UV excitation. The corresponding gas concentrations (C_ppm_) were calculated using the following formula (Wang et al., 2025):

(1) $$C_{ppm}=\frac{v_{\mu L}\times \rho_{mg/L}\times \omega }{M_{g/mol}\times V_{L}}\times 22.4\times 10^{-9}$$

where *v*_*μL*_ is the analyte solution volume, *ρ*_*mg/L*_ is the analyte solution density, *ω* is the mass fraction of the analyte solution, *M*_*g/mol*_ is the molecular weight of the analyte solution, and *V*_*L*_ is the volume of the test chamber.

### 2.5 Application of the ratiometric tag in shrimp freshness

Fresh shrimp (65 ± 5 g) were placed in the Petri dish. The fabricated ratiometric tag was affixed to the inside top of the sealed Petri dish, ensuring no direct contact with the sample. The samples were monitored at 4°C, during which the fluorescence colour changes of the ratiometric tag were captured using a smartphone camera under 365 nm UV excitation. For comparison, the freshness of the samples stored at 4°C was validated using TVB-N according to the national standards of the P.R. China (GB 5009.228-2016, GB 2707-2016). The freshness of shrimp can be classified into three grades based on TVB-N values (mg/100 g): TVB-N ≤ 15 for fresh, 15 < TVB-N < 30 for less fresh, and TVB-N ≥ 30 for spoilage (Chen et al., 2025). Furthermore, in accordance with the national standards of the P.R. China (GB 4789.2-2022), total viable counts (TVC) were determined and expressed as log_10_ CFU/g; typically, a TVC value of 6.0 log_10_ CFU/g is regarded as the spoilage threshold.

### 2.6 Image dataset construction

The dataset comprising 1842 independent samples (615 fresh, 609 less fresh, and 618 spoiled) was constructed to capture images of freshness-related conditions at different storage times. All samples were acquired under standard conditions. For CNNs training, no preprocessing was required for the obtained images. Data augmentation techniques, including flipping, rotation, scaling, cropping, translation, and Gaussian noise, were applied to enhance dataset diversity and improve model performance and generalization (Lin et al., 2024). In addition, an independent test set of 122 fluorescence images, separate from 1842-image dataset, was used for final model evaluation.

### 2.7 Training of CNNs

In this work, five representative CNN architectures (ResNet-34, VGGNet-16, AlexNet, GoogLeNet, and DenseNet) were used as candidate models. These selected architectures possessed a variety of network types, from basic sequential layers to complex residual and dense connections. This approach enabled a systematic comparison of predictive accuracy and deployment feasibility. Under the transfer learning strategy, each model was initialized using pre-trained weights. Simultaneously, the original classification heads of the model were replaced with a fully connected layer with three output nodes, designed to match the three freshness categories. During training, the cross-entropy loss function was adopted, and the Adam optimizer was utilized with an initial learning rate of 0.001. Training was performed for up to 100 epochs with a batch size of 32. Early stopping (patience = 10) was applied to mitigate overfitting, terminating training once validation accuracy plateaued.

The trained CNN models were tested on 122 unknown images. The overall accuracy, weighted-average precision, weighted-average recall, weighted-average F1-score, and macro-average multiclass receiver operator curve (ROC)-AUC were calculated to assess the predictive performance of the model. Based on the initial model comparison, ResNet-34 was further evaluated using stratified 5-fold cross-validation on the 1842-image dataset. In each fold, approximately 80% of the data were used for training and 20% for validation while maintaining comparable class proportions.

### 2.8 Statistical analysis

All experiments were repeated in triplicate, and the obtained results were analyzed using OriginPro 8.5 software (OriginLab Co., USA). Principal component analysis (PCA) was performed using the Metware Cloud, a free online platform for data analysis (https://cloud.metware.cn).

## 3 Results and discussion

### 3.1 Structural characterization of DPA-AgCuNCs

**Fig. 1:**
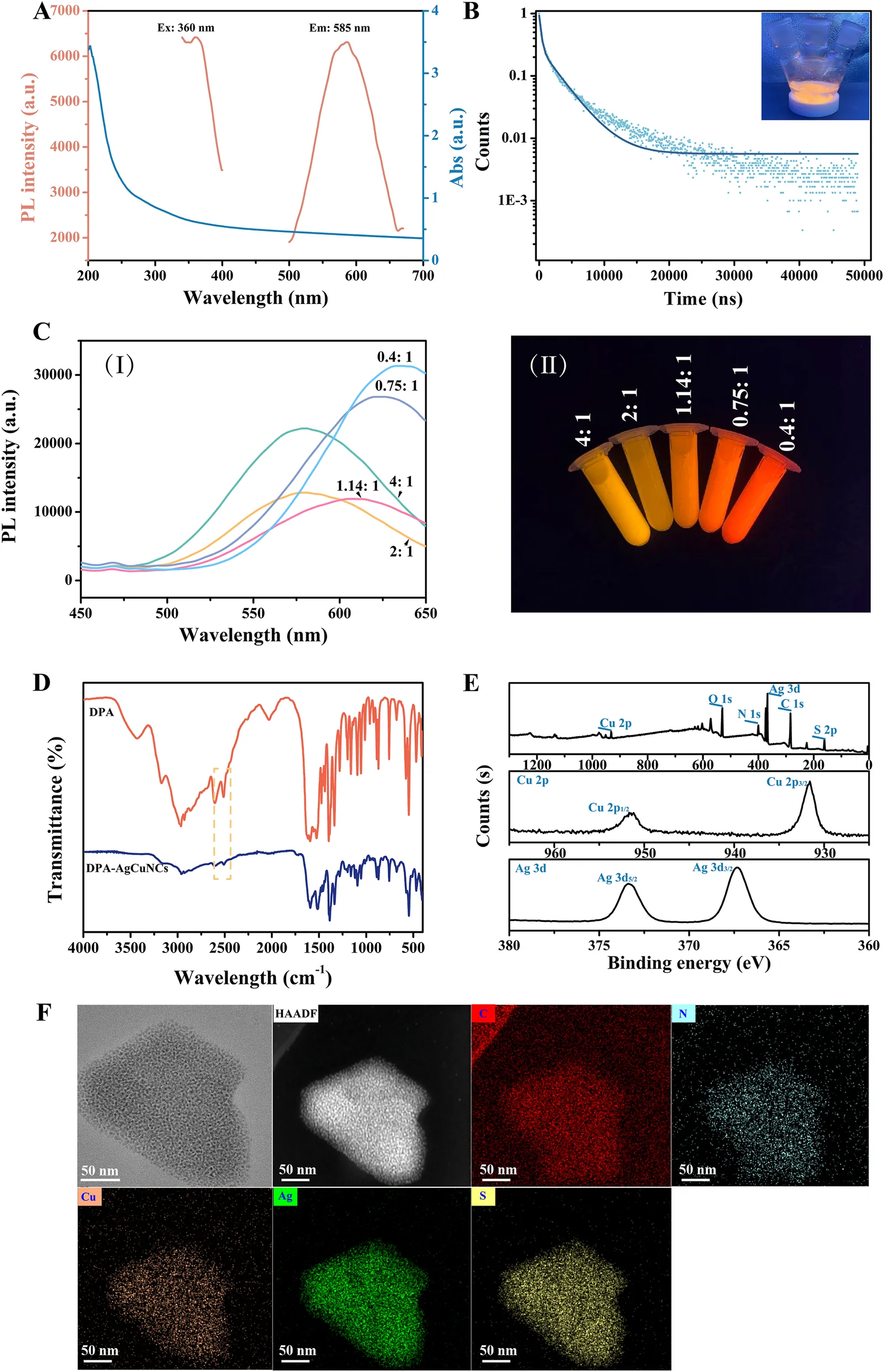
(A) The UV-vis absorption (blue line) and PL excitation and emission (orange line) spectra of DPA-AgCuNCs. (B) The PL decay curves of the DPA-AgCuNCs (inset: the photograph of the DPA-AgCuNCs solution under UV light). (C) The PL emission spectra (I) and photograph (II) of the DPA-AgCuNCs at different Cu^2+^/Ag^+^ ratios. (D) IR spectra of DPA (red line) and DPA-AgCuNCs (blue line). (E) XPS survey spectrum (top), Cu 2p region (middle), and Ag 3d region (bottom) of DPA-AgCuNCs. (F) TEM and EDX mapping of DPA-AgCuNCs.

In this work, DPA-AgCuNCs were synthesized via a microwave-assisted method at 90°C and 700 W for 150 s, followed by simple purification (Scheme 1A). The optical properties of the as-synthesized DPA-AgCuNCs were investigated using UV-vis absorption, photoluminescence (PL), and time-resolved PL spectroscopy. As shown in Fig. 1A, the absorption spectrum exhibited a featureless, monotonic increase in signal toward shorter wavelengths. Upon excitation at 360 nm, the DPA-AgCuNCs exhibited an emission peak at 585 nm (red line in Fig. 1A). Remarkably, intense orange luminescence was observed from precipitated DPA-AgCuNCs, demonstrating their AIE properties (inset in Fig. 1B). Time-resolved PL decay curves of the DPA-AgCuNCs were shown in Fig. 1B. According to the decay curves, the average PL lifetime (τ_ave_) was measured to be 4.023 μs. The observed large Stokes shift (225 nm) and long τ_ave_ (4.023 μs) indicated that the fluorescence originated from a ligand-to-metal charge transfer (LMCT) from the sulfur atom in DPA to the Ag/Cu core (Chang et al., 2024). Additionally, as depicted in Fig. 1C, the PL emission maxima of the as-synthesized DPA-AgCuNCs red-shifted from 585 to 630 nm with increasing proportion of Cu^2+^. Taken together, these results indicated that the fluorescence of DPA-AgCuNCs could be attributed to the LMCT from the sulfur atom in DPA to the Cu core.

From the FTIR spectra Fig. 1D, DPA showed a characteristic peak at 2509 cm^−1^, corresponding to the stretching vibration of -SH. This peak disappeared in the spectrum of DPA-AgCuNCs, indicating that DPA was successfully anchored to Cu/Ag core via the formation of Cu/Ag-S bond. The elemental composition and morphology of the DPA-AgCuNCs were further analyzed using XPS and TEM, respectively. Fig. 1E showed six dominant peaks with binding energies at 931.76, 530.11, 400.18, 367.33, 283.65, and 160.92 eV corresponding to Cu 2p, O 1s, N 1s, Ag 3d, C 1s, and S 2p, respectively. Furthermore, the valence states of the Cu and Ag were further investigated by high-resolution XPS. The Cu 2p spectrum displayed two peaks at 952.5 and 931.6 eV, corresponding to Cu 2p_1/2_ and 2p_3/2_, respectively. No satellite feature for Cu(Ⅱ) was observed, indicating that copper existed primarily in low oxidation states (Cu(0) or Cu(Ⅰ)) in the DPA-AgCuNCs (Han et al., 2018; Zhang et al., 2023). Similarly, the Ag 3d spectrum exhibited two symmetric peaks at 373.3 and 367.3 eV, assigned to Ag 3d_5/2_ and Ag 3d_3/2_ of Ag (0) (Ling et al., 2020). The morphology and elemental distribution of the DPA-AgCuNCs were further characterized by TEM and energy-dispersive X-ray spectroscopy (EDX). Fig. 1F showed that the DPA-AgCuNCs formed dense aggregates consisting of small nanoparticles with an average diameter of 2.99 ± 0.54 nm. EDX mapping confirmed the homogeneous spatial distribution of C, N, Cu, Ag, and S throughout the DPA-AgCuNCs, consistent with the elemental composition inferred from XPS analysis. Consequently, the above results demonstrated the successful synthesis of DPA-AgCuNCs.

### 3.2 Fabrication of ratiometric tag

**Fig. 2:**
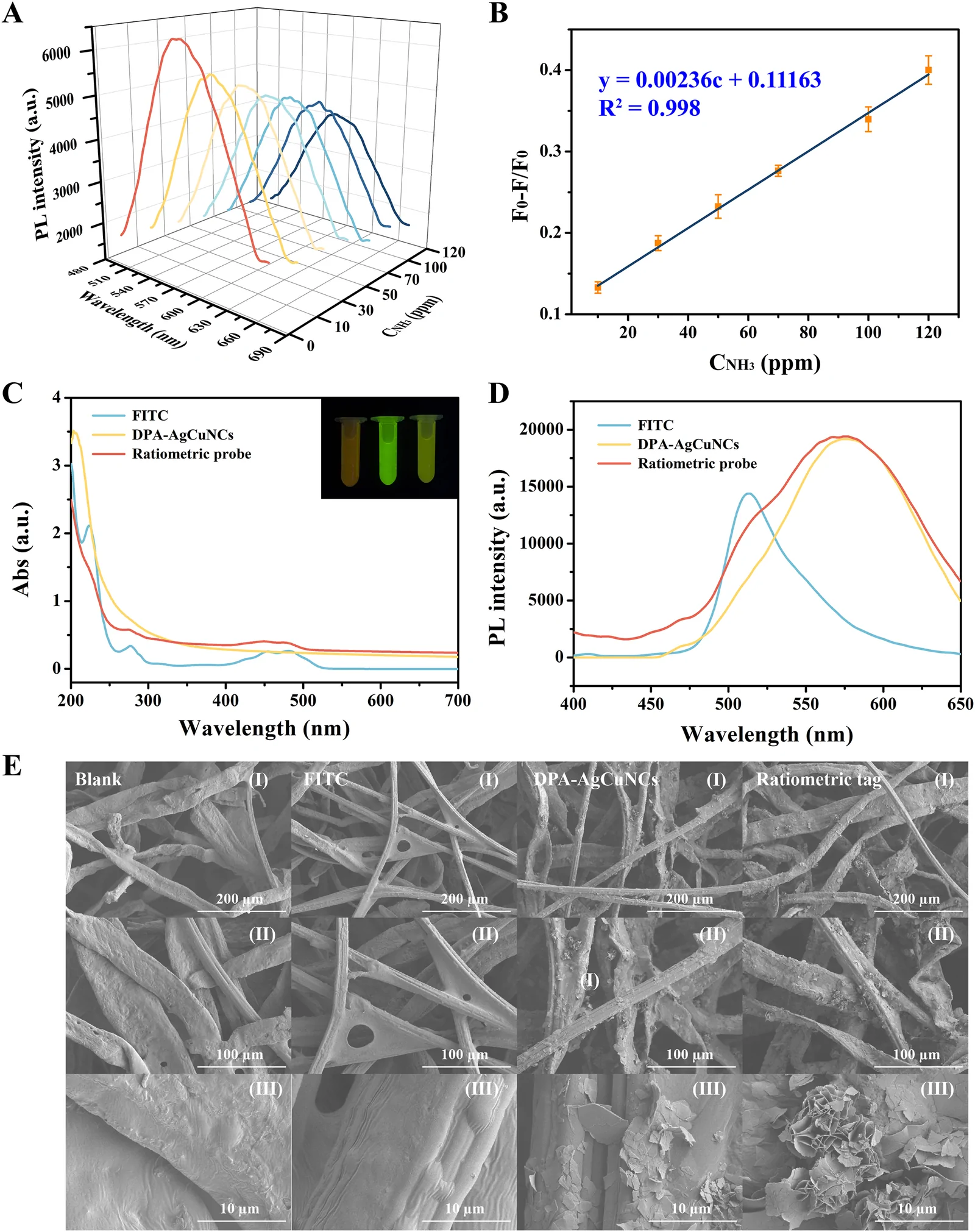
The fluorescent response of DPA-AgCuNCs to different concentrations of NH_3_, including (A) the recorded fluorescence spectra and (B) the calibration curve acquired from F_0_-F/F_0_ as a function of concentrations. F_0_ and F were the PL intensities at 585 nm of DPA-AgCuNCs with or without NH_3_, respectively. The UV-vis absorption (C) and PL emission (D) spectra of FITC (blue line), DPA-AgCuNCs (yellow line), and the ratiometric probe (red line). (E) SEM images of cellulose fiber paper, the loaded FITC tag, the loaded DPA-AgCuNCs tag, and the ratiometric fluorescent tag under different magnifications: (I) 250× (scale bar 200 μm), (II) 500 × (scale bar 100 μm), and (III) 5.0 k (scale bar 10 μm).

Before fabricating the ratiometric tag, the NH_3_-responsive behavior of the DPA-AgCuNCs was evaluated. As depicted in Fig. 2A, the PL intensity of DPA-AgCuNCs at 580 nm decreased significantly with increasing NH_3_ concentration. A linear relationship between the PL quenching efficiency and NH_3_ concentration was established in the range of 10 to 120 ppm (R^2^ = 0.998, Fig. 2B). The limit of detection was calculated to be 2.95 ppm (3σ/s), indicating good potential for NH_3_ detection.

Leveraging the intrinsic pH responsiveness of FITC, a ratiometric probe was constructed by integrating FITC with the NH_3_-sensitive DPA-AgCuNCs. As shown in Fig. 2C, the absorption spectrum of the resulting probe (red line) closely overlapped with the combined spectra of FITC (blue line) and DPA-AgCuNCs (yellow line), with no new absorption bands observed, indicating the absence of chemical interactions and confirming a physical mixing process. The PL emission peaks of the ratiometric probe (510 and 585 nm, red line in Fig. 2D) closely matched the superimposed emissions of FITC (510 nm) and DPA-AgCuNCs (585 nm), further corroborating the UV-vis results. Subsequently, the ratiometric probe was drop-cast onto fiber paper to prepare the ratiometric tag, and its surface morphology was characterized using SEM. As depicted in Fig. 2E, the pristine cellulose fiber paper displayed a porous fibrous architecture. After depositing FITC, large sheet-like aggregates were observed within the inter-fiber gaps. In contrast, the surface of the DPA-AgCuNCs-modified tag was decorated with abundant petal-like particles. SEM analysis of the co-deposited sample exhibited a mixture of sheet-like and petal-shaped particles on the fibrous network, confirming the successful integration of both components onto the substrate. Overall, these results confirmed the successful fabrication of the ratiometric tag.

### 3.3 Ammonia-responsive behaviour

**Fig. 3:**
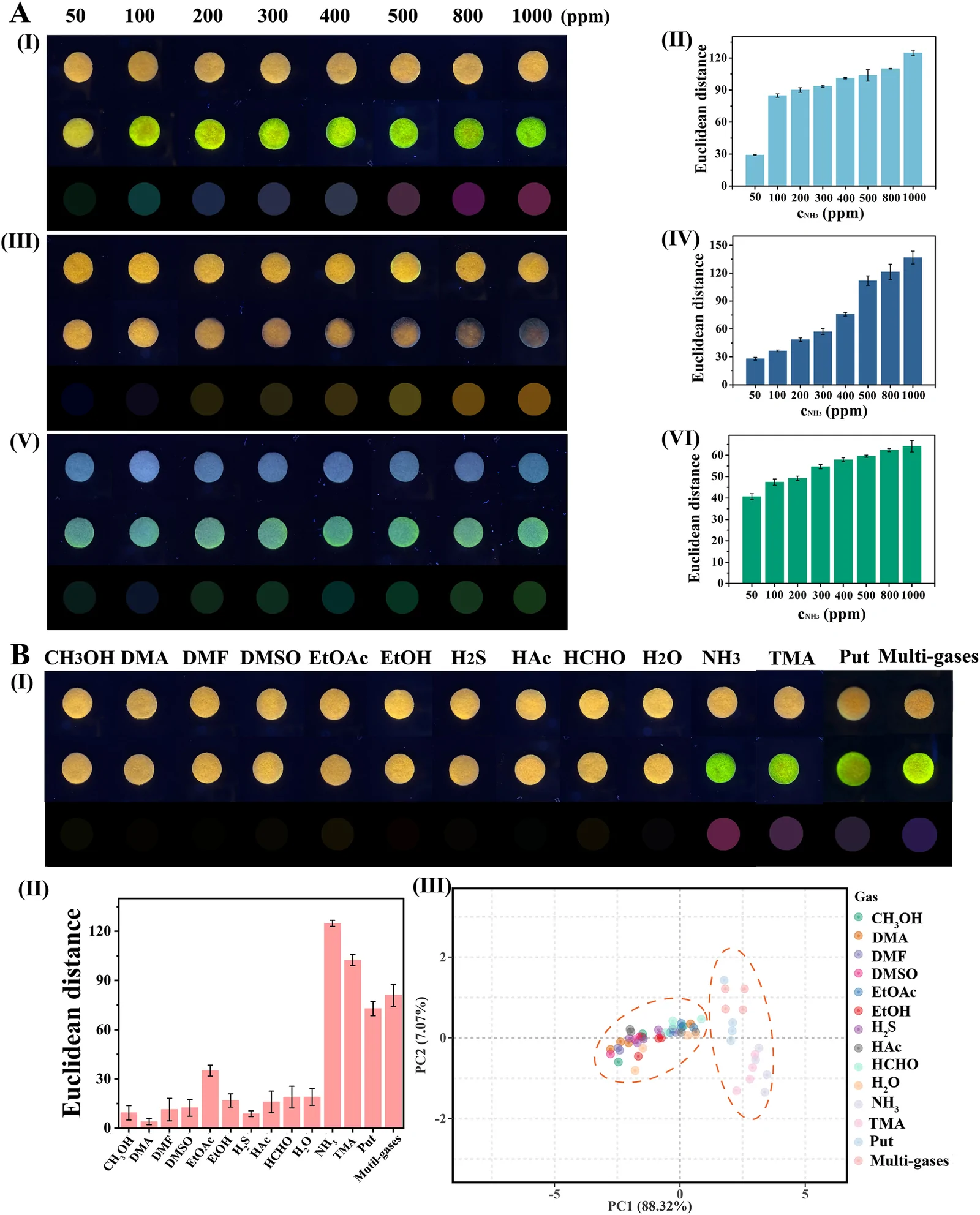
(A) Characteristic maps and Euclidean distance (ED) plot of ratiometric fluorescent tag (I –II), the loaded DPA-AgCuNCs tag (III – IV), and the loaded FITC tag (V – VI) after treating with different concentrations of NH_3_ (50 – 1000 ppm). (B) Characteristic maps (I), ED plot (II), and principal component analysis (PCA) score plot of ratiometric fluorescent tag after treating with different analytes (CH_3_OH, DMA, DMF, DMSO, EtOAc, EtOH, H_2_S, HAc, HCHO, H_2_O, NH_3_, TMA, Put, and multi-gases (NH_3_, TMA, H_2_S, and HCHO)).

After preparation, the fluorescence responses of three tags (loaded DPA-AgCuNC tag, the loaded FITC tag, and the ratiometric tag) toward NH_3_ were systematically explored under simulated gas conditions. As displayed in Fig. 3A(Ⅰ), the ratiometric tags exhibited a pronounced fluorescent colour change from orange-yellow to dark green with increasing NH_3_ concentration under 365 nm UV excitation. Furthermore, the colour variations observed in the difference images became increasingly pronounced as the NH_3_ concentration increased. This enhanced contrast was quantitatively supported by a corresponding increase in Euclidean distance (ED) values (Fig. 3A(Ⅱ)), confirming its feasibility for semi-quantitative monitoring of NH_3_. For comparison, the fluorescence colour responses of the loaded DPA-AgCuNCs tag (Fig. 3A(Ⅲ)) and the loaded FITC tag (Fig. 3A(Ⅴ)) were also studied. In contrast to the ratiometric tag, the loaded DPA-AgCuNCs tag and the loaded FITC tag exhibited less pronounced colour variations. However, their difference images and corresponding ED values still displayed an increasing trend with rising ammonia concentrations. Benefiting from its dual-colour transitions and high ED value, the fabricated ratiometric tag possessed excellent visual discrimination and enhanced sensing performance for NH_3_ monitoring.

In addition, the selectivity of the ratiometric tag was evaluated using 13 volatile substances (i.e., CH_3_OH, DMA, DMF, DMSO, EtOAc, EtOH, H_2_S, HAc, HCHO, H_2_O, TMA, Put NH_3_) produced during seafood spoilage. As depicted in Fig. 3B, the ratiometric tag emitted orange-yellow fluorescence under 365 nm UV excitation. Exposure to most of the tested interferents caused only negligible changes in the corresponding difference images, whereas pronounced responses were observed for NH_3_, TMA, Put. To further evaluate the sensing performance under a more complex volatile environment, a multicomponent system containing NH_3_, TMA, H_2_S, and HCHO was also investigated. A distinct fluorescence response was maintained in the presence of the mixed volatiles, indicating that the tag was capable of responding to representative spoilage related compounds even under multicomponent conditions. As shown in Fig. 3B(Ⅱ), the ED values obtained for NH_3_, TMA, Put, and a multicomponent system were significantly higher than those of other gases. Furthermore, the PCA score analysis (Fig. 3B(Ⅲ)) revealed that NH_3_, TMA, Put, and a multicomponent system were clearly separated from the majority of the tested interferents, further confirming the distinct response characteristics of the ratiometric tag toward representative spoilage-related volatile markers.

### 3.4 Sensing mechanism exploration

**Fig. 4:**
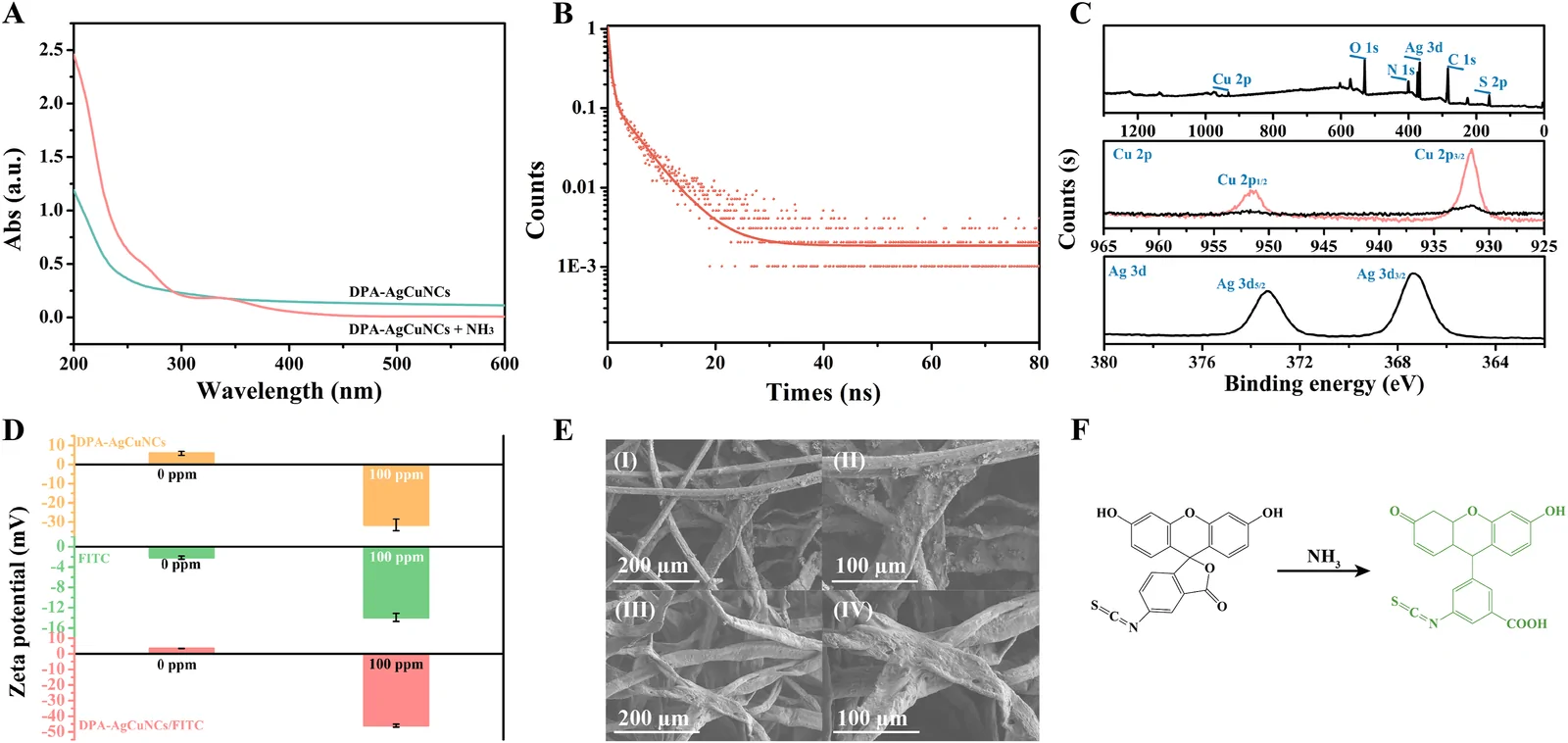
Mechanistic study of DPA-AgCuNCs for detecting NH_3_. UV-Vis (A), time-resolved PL decay spectra (B), XPS survey spectrum, Cu 2p region, and Ag 3d region (C), and zeta potential (D) of DPA-AgCuNCs after exposure to NH_3_. (E) SEM images acquired before (Ⅰ and Ⅱ) and after (Ⅲ and Ⅳ) NH_3_ exposure. (F) Mechanism diagram of FITC after exposure to NH_3_.

Following the evaluation of sensitivity and selectivity, the sensing mechanism of the ratiometric tag was explored using UV-vis, PL lifetime, XPS, zeta (ζ) potential, and TEM analysis. As shown in Fig. 4A, the UV-vis absorption spectra of DPA-AgCuNCs before exposure to NH_3_ exhibited a featureless profile with a monotonic increase toward shorter wavelengths. After exposure to NH_3_, two new absorption bands appeared at 266.8 and 338.1 nm. This distinct spectral change provided direct evidence for the formation of a new, nonfluorescent ground-state complex, consistent with a static fluorescence quenching mechanism. The time-resolved PL decay analysis (Fig. 4B) revealed a significant decrease in the τ_ave_ from 4.023 μs (DPA-AgCuNCs) to 3.27 ns after exposure to NH_3_. Such an orders-of-magnitude reduction in τ_ave_ was indicative of dynamic fluorescence quenching.

As depicted in Fig. 4C, XPS quantitative analysis after NH_3_ exposure yielded atomic composition of Cu (0.79%), O (20.14%), N (10.49%), Ag (3.64%), C (56.02%), and S (8.93%). Notably, the nitrogen content increased from 9.13% to 10.49%, indicating the incorporation of NH_3_ into the system. In addition, significant changes were observed in the Cu 2p spectra: the characteristic sharp doublets associated with Cu(I) in DPA-AgCuNCs disappeared after NH_3_ exposure (blank line in Fig. 4C), while broadened features emerged, indicating a change in copper oxidation state or coordination environment. Taken together, XPS and UV-vis results indicated the formation of a substance upon reaction with NH_3_, likely an NH_3_-coordinated Cu/Ag complex, which exhibited electronic transition characteristics distinct from those of DPA-AgCuNCs.

The zeta (ζ) potentials of the DPA-AgCuNCs, FITC, and ratiometric probe were measured before and after exposure to NH_3_. As depicted in Fig. 4D, the absolute zeta potentials of all three samples increased after NH_3_ exposure, suggesting enhanced colloidal stability (Zhang et al., 2023). SEM images acquired after NH_3_ exposure revealed the disaggregation of the DPA-AgCuNCs aggregates (Fig. 4E). The morphological changes, along with the changes in the UV-vis spectrum, XPS, and zeta potential, provided strong evidence for the formation of a non-fluorescent ground-state complex between DPA-AgCuNCs and NH_3_, supporting the static quenching mechanism. Taken together with the substantial decrease in PL lifetime, the overall fluorescence quenching was attributed to a synergistic static-dynamic quenching mechanism, with static quenching as the predominant pathway.

Furthermore, the sensing mechanism of FITC toward NH_3_, as illustrated in Fig. 4F, has been verified in previous studies (Mu et al., 2025; Wang et al., 2021, 2025). Typically, in the presence of NH_3_, the lactone ring of FITC opens to form a carboxyl group (Wang et al., 2025), forming pH-responsive green fluorescence. Leveraging this property, FITC was ratiometrically integrated with the orange-emitting DPA-AgCuNCs, thereby constructing a sensitive dual-signal sensing platform.

### 3.5 Optimization of ratiometric tag

**Fig. 5:**
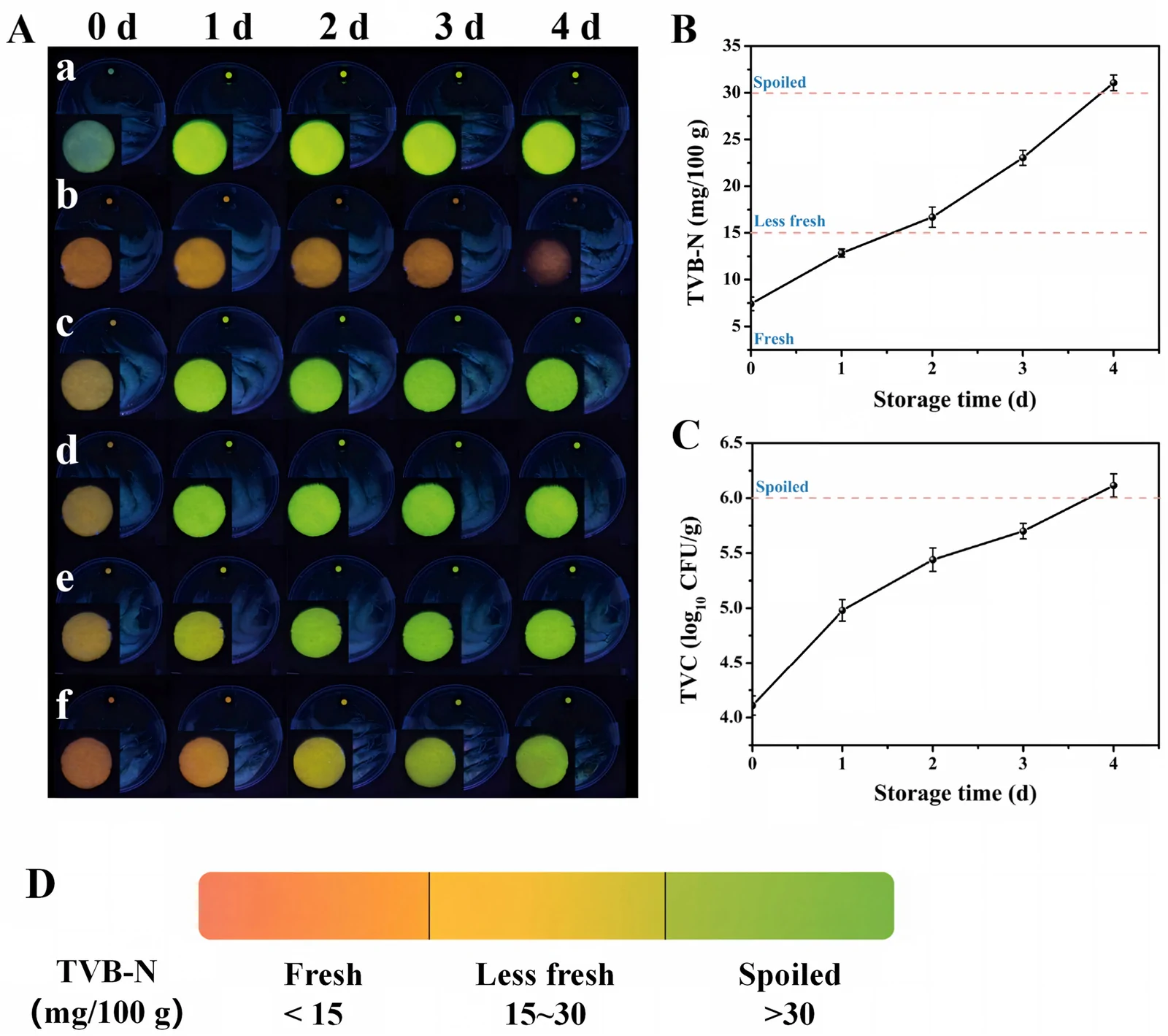
(A) The optimization of the volume ratio of DPA-AgCuNCs and FITC for the fabricated ratiometric fluorescent tag, and monitoring shrimp freshness using these tags (a: the loaded FITC tag, b: the loaded DPA-AgCuNCs tag, c: *v*_DPA-AgCuNCs_: *v*_FITC_ = 2.5: 2, d: *v*_DPA-AgCuNCs_: *v*_FITC_ = 3: 1.5, e: *v*_DPA-AgCuNCs_: *v*_FITC_ = 3.5: 1, f: *v*_DPA-AgCuNCs_: *v*_FITC_ = 4: 0.5). The changes of TVB-N (B) and TVC (C) in shrimp. (D) The designed fluorescence colour card used to assess the shrimp freshness based on the fluorescence response of Fig. 5A (f: *v*_DPA-AgCuNCs_: *v*_FITC_ = 4: 0.5).

After confirming the sensing performance toward NH_3_, the practical applicability of the ratiometric tag was evaluated for seafood freshness monitoring using shrimp as a representative model. The volume ratio of DPA-AgCuNCs to FITC (*v*_DPA-AgCuNCs_: *v*_FITC_) was optimized to achieve optimal sensing performance. As depicted in Fig. 5A, the loaded FITC tag (group a) exhibited weak green emission, whereas the loaded DPA-AgCuNCs tag (group b) displayed orange fluorescence. After mixing, the fluorescence colour of the ratiometric tag gradually changed from light-yellow to orange-yellow and finally to orange with increasing volume fraction of DPA-AgCuNCs (groups c – f).

Subsequently, six groups of tags (groups a – f) with different volume ratios of DPA-AgCuNCs to FITC were fabricated to evaluate shrimp freshness under refrigerated conditions. Standardized freshness parameters, including TVB-N (Fig. 5B) and TVC (Fig. 5C), were measured to verify the accuracy of these tags. As storage time prolonged, the gradual increase in TVB-N and TVC values clearly reflected the transition from fresh to spoiled states. Based on the obtained results, shrimp samples were classified into fresh (0 – 1 day), less fresh (2 – 3 days), and spoiled (4 days), providing a reliable reference for evaluating the sensing performance.

Fig. 5A showed that group a emitted weak green fluorescence. After 1 day of storage, the colour of the tag gradually shifted to light green. In contrast, group b exhibited strong orange emission during the initial stage. As the storage time prolonged, its fluorescence intensity gradually decreased. After 4 days of storage, the fluorescence was almost completely quenched. At this point, both the TVB-N and TVC values exceeded the corresponding thresholds, indicating that the shrimp had spoiled. Overall, groups a and b exhibited relatively monotonous fluorescence colour changes during storage, making them unsuitable for visual assessment.

As storage time increased, groups c – f showed distinct changes in fluorescence colour. Although groups c – e exhibited visible fluorescent colour changes, these changes could not be consistently correlated with the corresponding freshness levels determined by TVB-N and TVC values. In comparison, group f initially showed a bright orange emission. As storage time increased, the fluorescence colour of the tag gradually changed from bright orange to pale yellow, corresponding to a decline in shrimp freshness from fresh to less fresh, and eventually turned green when shrimp became spoiled. Thus, group f (*v*_DPA-AgCuNCs_: *v*_FITC_ = 4: 0.5) was used for subsequent studies.

Based on the distinct fluorescence colour changes of the tag (group f) during shrimp storage, a fluorescence colour card was further established to enable direct naked-eye differentiation among fresh, less fresh, and spoiled samples (Fig. 5D). This colour card provides a rapid and intuitive means for preliminary freshness screening by visual comparison, while CNN-assisted analysis can be further employed for more objective and standardized classification.

Furthermore, the environmental stability of the fabricated tag was further evaluated under refrigerated storage, different relative humidities, and 365 nm UV exposure (Fig. S1). After 30 days, the ratiometric tag retained 94.47% of its initial fluorescence at 4°C and more that 91% under approximately 33%∼90% relative humidity at room temperature. Moreover, 95.47% of initial fluorescence was retained after exposure to 365 nm UV light for 5 days. These results demonstrated satisfactory stability under the investigated environmental conditions.

### 3.6 Analysis of the performance of the CNN-assisted sensing platform

To minimize the negative effects of environmental interference and subjective misjudgment, a CNN-assisted fluorescence sensor integrated with a portable ratiometric fluorescent monitoring system was proposed for rapid assessment of seafood freshness. In this study, five representative CNN architectures, including ResNet-34, VGGNet-16, AlexNet, GoogLeNet, and DenseNet, were used for freshness classification. To conduct fully supervised training, a dataset comprising 1842 labeled images was used as the primary training set.

As shown in Table 1, ResNet-34 demonstrated the best overall performance, achieving an overall accuracy of 98.39%, an average precision of 98.48%, an average recall of 98.41%, an average F1-score of 98.41%, and an AUC of 99.02%. For comparison, VGGNet-16 and DenseNet performed moderately, with an overall accuracy of 75.00% and 76.27%, respectively. GoogLeNet showed relatively weaker predictive performance, with an overall accuracy of 53.48% and an AUC of 75.79%. Unfortunately, AlexNet exhibited the poorest performance, with an overall accuracy of 36.08% and an F1-score of 17.67%, indicating its limited ability to extract features from fluorescence images. These results demonstrated ResNet-34 as the optimal architecture among the evaluated models.

**Table 1:** Comparison of the classification effects of different models.

Models	Accuracy	Precision	Rcall	F1-score	AUC
VGGNet-16	0.7500	0.7204	0.7323	0.7500	0.9210
AlexNet	0.3608	0.1203	0.3333	0.1767	0.5577
GoogLeNet	0.5348	0.6986	0.4397	0.4657	0.7579
DenseNet	0.7627	0.7760	0.7343	0.7710	0.8986
ResNet-34	0.9839	0.9848	0.9841	0.9841	0.9902

Based on its superior performance in the initial model comparison, ResNet-34 was further evaluated using stratified 5-fold cross-validation to assess its robustness across different data partitions. As shown in Table S1, across the five folds, the model achieved an accuracy of 96.81 ± 0.92%, an average precision of 96.82 ± 0.92%, an average recall of 96.81 ± 0.92%, an average F1-score of 96.81 ± 0.92%, and an AUC of 99.58 ± 0.26%. The small standard deviations across the five folds, particularly 0.92% for accuracy and 0.26% for AUC, together with consistently high performance in all folds, indicated that the model was relatively insensitive to different data partitions and exhibited stable classification performance.

**Fig. 6:**
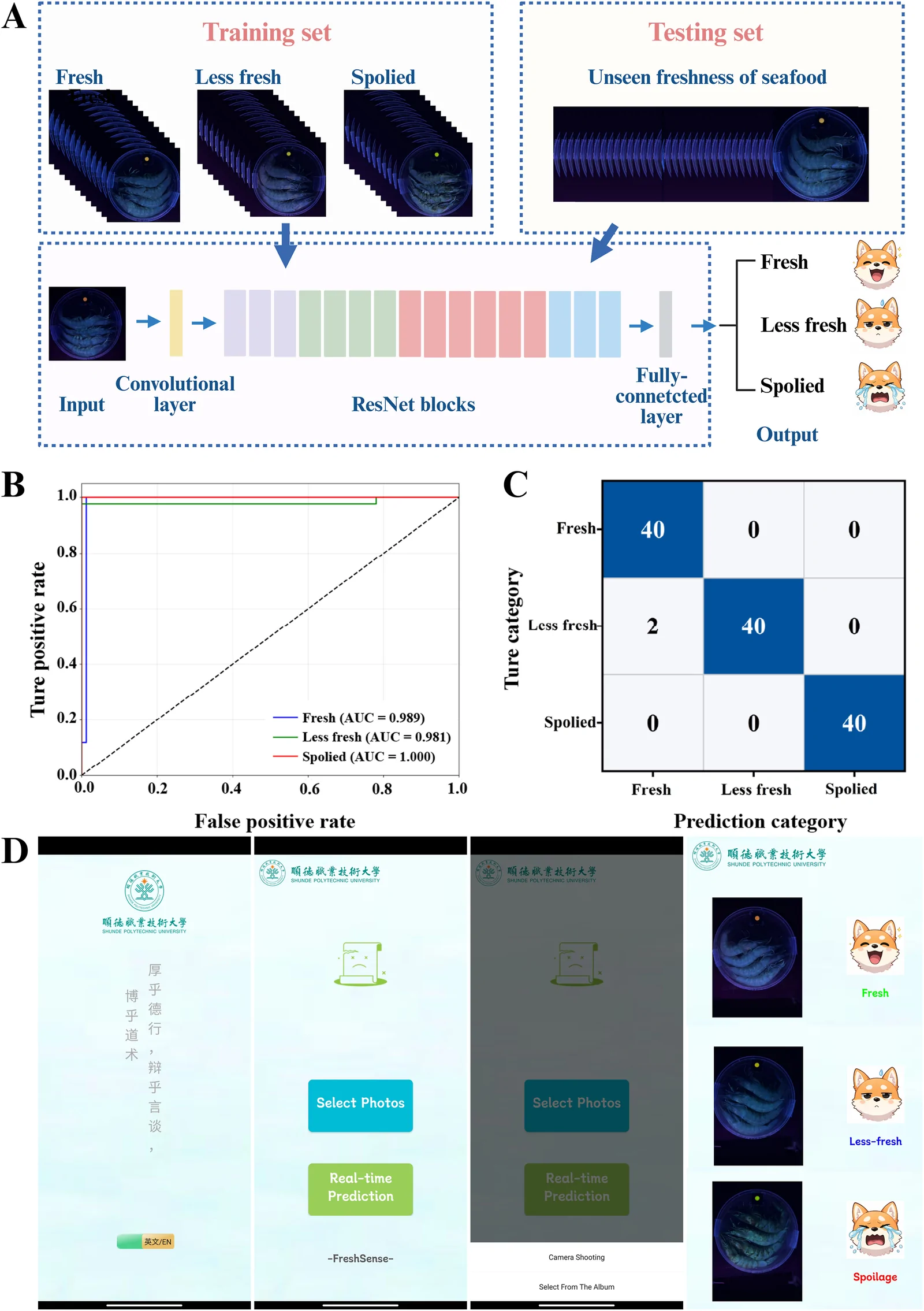
(A) Schematic diagram of the CNN model used for shrimp freshness classification. The receiver operating characteristic (ROC) curve (B) and confusion matrix (C) for the testing set across three freshness levels of the ResNet-34 model. (D) Smartphone APP interface presentation.

To further assess its generalization capability, the trained ResNet-34 was evaluated using a test set comprising 122 unknown images (Fig. 6A). The ROC curve was used to study the classification performance of the ResNet-34 model. As shown in Fig. 6B, the ROC curve was well above the random baseline, with an AUC value of 99.02%, demonstrating excellent classification performance and robustness. In addition, the confusion matrix (Fig. 6C) showed that most samples were correctly classified, with only two misclassified samples observed in the fresh group. Based on the 122 tested images, the model achieved precision values of 100% for fresh, 95.24% for less fresh, and 100% for spoiled samples.

Although the tag response can be visually interpreted using the fluorescence colour card, CNN-assisted analysis improves the objectivity and consistency of freshness classification, especially for intermediate colour responses. To automate the overall screening procedure, the optimized ResNet-34 architecture was incorporated in a smartphone APP (FreshSense). As shown in Fig. 6D, consumers can use the APP directly to capture fluorescence images of ratiometric fluorescent tags, and the APP automatically processes the input data and displays the resulting freshness prediction on the screen. This integration demonstrated the feasibility of combing CNN-based image analysis with portable fluorescent sensing for rapid on-site food freshness assessment.

To further evaluation the applicability of the developed sensing system across different seafood matrices, salmon, cod, and grass carp were additionally investigated at 4°C (Fig. S2). The classification accuracies for salmon, cod, and grass carp were 93.52%, 90.74%, and 92.59%, respectively. These results demonstrated that the developed sensing system maintains satisfactory freshness classification performance across different seafood matrices.

It should be noted that the current smartphone application represents a proof-of-concept implementation in which model inference is performed on a remote server. Future work will focus on model compression and on-device deployment, together with systematic evaluation of computational and operational costs, to reduce network dependence and further improve the practicality of fully offline freshness assessment.

## 4 Conclusion

In summary, this study developed a ratiometric sensing platform for rapid seafood freshness assessment by integrating orange-emitting DPA-AgCuNCs with AIE properties and green-emissive FITC. The two fluorescent probes exhibited distinct and complementary fluorescence responses to NH_3,_ in which DPA-AgCuNCs were quenched through combined static and dynamic quenching mechanism, while FITC fluorescence was enhanced through conversion its carboxylate form, resulting in a distinct orange-to-green fluorescence transition. Based on this visual response, a fluorescence colour card was established to enable rapid preliminary freshness screening by naked-eye comparison. CNN-assisted image analysis was further introduced as a complementary approach to improve the objectivity and consistency of freshness classification, achieving an overall accuracy of 98.36% for shrimp samples. The applicability of the developed system was further demonstrating using salmon, cod, and grass carp, with classification accuracies of 93.52%, 90.74%, and 92.59%, respectively. The optimized ResNet-34 architecture was integrated into smartphone APP as a proof-of-concept implementation, demonstrating the feasibility of combing portable fluorescent sensing with automated freshness classification. Further work will focus on model compression, on-device deployment, and systematic evaluation of computational and operational costs to improve the practicality of fully offline freshness assessment.

## CRediT authorship contribution statement

Wenyang Zhang: Conceptualization, Methodology, Investigation, Formal analysis, Writing - original draft, Project administration, Funding acquisition. You Tian: Writing - review & editing, Data curation, Visualization. Yanwu Chen: Supervision. Min Wei: Software, Methodology.

## Data availability

Data will be made available on request.

## Declaration of competing interest

The authors declare that they have no known competing financial interests or personal relationships that could have appeared to influence the work reported in this paper.
