# Supplementary material for: CNN-integrated ratiometric fluorescent tag for rapid monitoring of seafood freshness at refrigerated temperatures
Source: Curr Res Food Sci. 2026 Sep 8;13:101560. doi: 10.1016/j.crfs.2026.101560 (PMC13579589; doi:10.1016/j.crfs.2026.101560)
Supplement: Supplementary file 1 [file mmc1.docx]

**Supporting Information**

**CNN-integrated ratiometric fluorescent tag for rapid monitoring of seafood freshness at refrigerated temperatures**

Wenyang Zhang ^a,*^, You Tian ^b, c, d^, Yanwu Chen ^a^, Min Wei ^e^

^a^College of Light Chemical Industry and Materials Engineering, Shunde Polytechnic University, Foshan, 528333, China

^b^School of Food Science and Engineering, South China University of Technology, Guangzhou 510641, China

^c^Academy of Contemporary Food Engineering, South China University of Technology, Guangzhou Higher Education Mega Centre, Guangzhou 510006, China

^d^Engineering and Technological Research Centre of Guangdong Province on Intelligent Sensing and Process Control of Cold Chain Foods, & Guangdong Province Engineering Laboratory for Intelligent Cold Chain Logistics Equipment for Agricultural Products, Guangzhou Higher Education Mega Centre, Guangzhou 510006, China

^e^College of Food Science and Technology, Henan Key Laboratory of Cereal and Oil Food Safety Inspection and Control, Henan University of Technology, Zhengzhou, 450001, China

* Corresponding author: [23097@sdpt.edu.cn](mailto:23097@sdpt.edu.cn) (W. Zhang)

**S2 Materials and methods**

**S2.5 Stability evaluation of the the** **ratiometric tag**

The environmental stability of the fabricated ratiometric tag was evaluated under different storage conditions. For refrigerated storage stability, the fabricated tags were stored at 4℃ for 30 days. To investigate the influence of environment humidity, different relative-humidity (RH) environments were established at room temperature using saturated salt solutions. Saturated MgCl_2_, NaBr, and KCl solutions were used to generate low-, medium, and high-humidity environments corresponding to approximately 33%, 60%, and 90% RH, respectively. The fabricated ratiometric tags were placed in sealed chambers above the saturated salt solutions without direct contact with the solutions and stored for 30 days. In addition, the photostability of the tag was evaluated by exposure to 365 UV light for 5 days. Three replicate tags were evaluated for each condition.

Fluorescence images were acquired at day 0 and after the specified storage or irradiation period under identical imaging conditions. For quantitative image analysis, and equal-sized circular region of interest (ROI) was selected from each fluorescent tag, while an adjacent dark region was used for background correction. The relative fluorescence intensity was calculated from the background-corrected RGB intensity according to Eq (s1):

$\text{I}\text{=}\frac{\text{(}\text{R}\text{−}\text{R}_{\text{bg}}\text{)+(}\text{G}\text{−}\text{G}_{\text{bg}}\text{)+(}\text{B}\text{−}\text{B}_{\text{bg}}\text{)}}{\text{3}}$	(s1)

where R, G, B represented the average red, green, and blue intensities of the fluorescent ROI, respectively, and R_bg_, G_bg_, and B_bg_ represented the corresponding intensities.

The fluorescence retention (R) was calculated using Eq (s2):

$\text{R}\text{(\%)=}\frac{\text{I}_{\text{t}}}{\text{I}_{\text{0}}}$	(s2)

where I0 and It represented the backgroup-corrected fluorescence intensities before and after storage or UV exposure, respectively.

**S3 Results and discussion**

S**3.5 Optimization of ratiometric tag**

The stability of the fabricated ratiometric tag was evaluated under refrigerated storage, different humidity conditions, and prolonged UV exposure (Figure S1). After storage at 4℃ for 30 days, the tag retained 94.97% of its initial fluorescence intensity, demonstrating satisfactory stability under the refrigerated conditions commonly used for seafood preservation.

The influence of environmental humidity was further investigated at room temperature. After 30 days of storage at approximately 33%, 60%, and 90% RH, the fluorescence retention values were 91.02%, 95.22%, and 93.00%, respectively. More than 91% of the initial fluorescence was retained throughout the investigated humidity range, and no pronounced deterioration of fluorescence was observed even under the high-humidity condition of 90% RH.

The fabricated ratiometric tag also exhibited satisfactory photostability. After exposure to 365 nm UV light for 5 days, 95.47% of the initial fluorescence was retained. Collectively, these results indicated that the ratiometric tags maintain relatively stable fluorescence under refrigerated storage, abroad range of environmental humidities, and prolonged UV exposure, supporting its potential use for seafood freshness monitoring under variable storage environments.

**Figure S1** The stability of the fabricated ratiometric tags under different environmental conditions: (A) 4℃; (B) room temperature, 33% RH; (C) room temperature, 60% RH; (D) room temperature, 90% RH; (E) 365 nm UV light.

**Figure S2** Validation of the developed APP for salmon (A), cod (B) and grass carp (C) stored at 4℃, (Ⅰ) the APP-based freshness prediction outputs, (Ⅱ) the statistical release of TVB-N over days, and (Ⅲ) confusion matrix of the tested samples.

**Table S1 Comparison of the classification effects of ResNet-34 evaluated using stratified 5-fold cross-validation**

| **Fold** | **Accuracy** | **Precision** | **Rcall** | **F1-score** | **AUC** |
| --- | --- | --- | --- | --- | --- |
| **1** | 0.9623 | 0.9626 | 0.9623 | 0.9624 | 0.9976 |
| **2** | 0.9565 | 0.9564 | 0.9564 | 0.9564 | 0.9964 |
| **3** | 0.9826 | 0.9827 | 0.9826 | 0.9826 | 0.9961 |
| **4** | 0.9652 | 0.9652 | 0.9652 | 0.9651 | 0.9908 |
| **5** | 0.9739 | 0.9742 | 0.9740 | 0.9739 | 0.9981 |
| **Average** | 0.9681 | 0.9682 | 0.9681 | 0.9681 | 0.9958 |
| **Std.** | 0.00917 | 0.00924 | 0.00921 | 0.00921 | 0.00259 |
